# Meta-analysis of laparoscopic vs. open resection of gastric gastrointestinal stromal tumors

**DOI:** 10.1371/journal.pone.0177193

**Published:** 2017-05-09

**Authors:** Liangying Ye, Xiaojing Wu, Tongwei Wu, Qijing Wu, Zhao Liu, Chuan Liu, Sen Li, Tao Chen

**Affiliations:** 1 The First Clinical Medical College of southern medical university, Guangzhou, Guangdong Province, China; 2 Department of Spinal Surgery, Affiliated Traditional Chinese Medicine Hospital, Southwest Medical University, Luzhou, China; 3 Department of General Surgery, Nanfang Hospital, Southern Medical University, Guangzhou, Guangdong Province, China; Virginia Mason Medical Center, UNITED STATES

## Abstract

**Background:**

This meta-analysis compared laparoscopic surgery (LAP) and open resection (OPEN) for the treatment of gastric gastrointestinal stromal tumors (GISTs) with regard to feasibility and safety.

**Methods:**

We searched PubMed, Embase, and Web of Science for studies published before March 2016 comparing the LAP and OPEN procedures for GISTs. RevMan 5.1 software was used for the meta-analysis.

**Results:**

In total, 28 studies met the inclusion criteria for the meta-analysis. The mean tumor sizes in the OPEN and LAP groups were 4.54 and 5.67 cm. Compared with the OPEN patients, the LAP patients experienced shorter surgical times (P = 0.05), less blood loss (P<0.01), earlier time to flatus (P<0.01) and an oral diet (P<0.01), and shorter hospital stays (P<0.01). The LAP patients also exhibited a decrease in overall complications (P<0.01). In addition, regarding the subgroup of larger GISTs (>5 cm), the present study did not report significant differences in operation time (P = 0.93), postoperative complications (P = 0.30), or recurrence rate (P = 0.61) between the two groups, though LAP was associated with favorable results regarding blood loss (P = 0.03) and hospital stay (P<0.01).

**Conclusions:**

Compared with the OPEN procedure, the LAP procedure is associated with preferable short-term postoperative outcomes and does not compromise long-term oncological outcomes. For gastric GISTs >5 cm, no significant difference was detected between LAP and OPEN if patient selection and intraoperative decisions were carefully considered.

## Introduction

Gastrointestinal stromal tumors (GISTs) are the most common mesenchymal tumors in the gastrointestinal tract (GI) [1]. GISTs can occur anywhere in the GI tract but are predominantly found in the stomach and small intestines, although they have also been reported in the omentum, mesentery and peritoneum [2]. Although mesenchymal tumors are thought to constitute only 1% of primary GI cancers [1, 3], the possibility of their occurrence should not be ignored.

Although tyrosine kinase inhibitors have led to considerable treatment success, surgical resection remains the most important component of treatment for resectable non-metastatic GISTs [4]. Open resection (OPEN) is the traditionally treatment for GISTs worldwide. However, since Lukaszczyk and Preletz first performed laparoscopic surgery (LAP) for GIST patients in 1992 [5], additional attention has been focused on this new technology, which offers the potential for reduced trauma and complications. However, the efficiency and safety of LAP for GISTs remain controversial and are affected by the surgeon’s laparoscopic skills and the technical feasibility of the procedure. To date, only a few small studies have compared LAP with OPEN for GISTs; however, the sample sizes of these studies were not sufficient. The present study aimed to systematically review the current literature comparing laparoscopic surgery to open resection for GISTs and to provide a comprehensive analysis of these techniques.

## Methods

Under C.T’s supervision, W.TW and Y.LY conducted a systematic search of the PubMed, Embase and Web of Science databases. The search terms included “gastrointestinal stromal tumor”, “GIST”, “laparoscope”, “gastrectomy” and “gastric resection”. We examined the titles and abstracts of potentially relevant articles and then retrieved the full texts of the articles for detailed review. The reference lists of articles that met the inclusion criteria of our analysis were scanned and searched for citations in the Web of Knowledge, Google Scholar and Google to obtain additional studies.

The inclusion criteria were as follows: (1) comparative peer-reviewed studies of LAP versus OPEN procedures; (2) human trials of patients with histologically confirmed GISTs; and (3) studies that mention at least one of the quantitative outcomes. Papers that contained any of the following were excluded: (1) tumors outside of the stomach, such as in the jejunum or ileum; (2) studies without a control group; and (3) studies without available data. If two studies by the same group were identified, the most recent study or the study that included more subjects was selected unless the reports were from different time periods.

Two authors, W.XJ and W.QJ, independently reviewed and extracted the required data using standard forms. The extracted data included the author, study period, geographical region, number of patients, operation time, blood loss, time to flatus, time to oral intake, length of hospital stay, morbidity, and long-term outcomes. Disagreements were resolved through discussions among the authors to achieve a consensus. Quality assessments were performed by Chen and Ye using the Newcastle–Ottawa scoring system as follows: studies with 5 to 9 stars were defined as high quality, and studies with <5 stars were defined as low quality.

**Fig 1:**
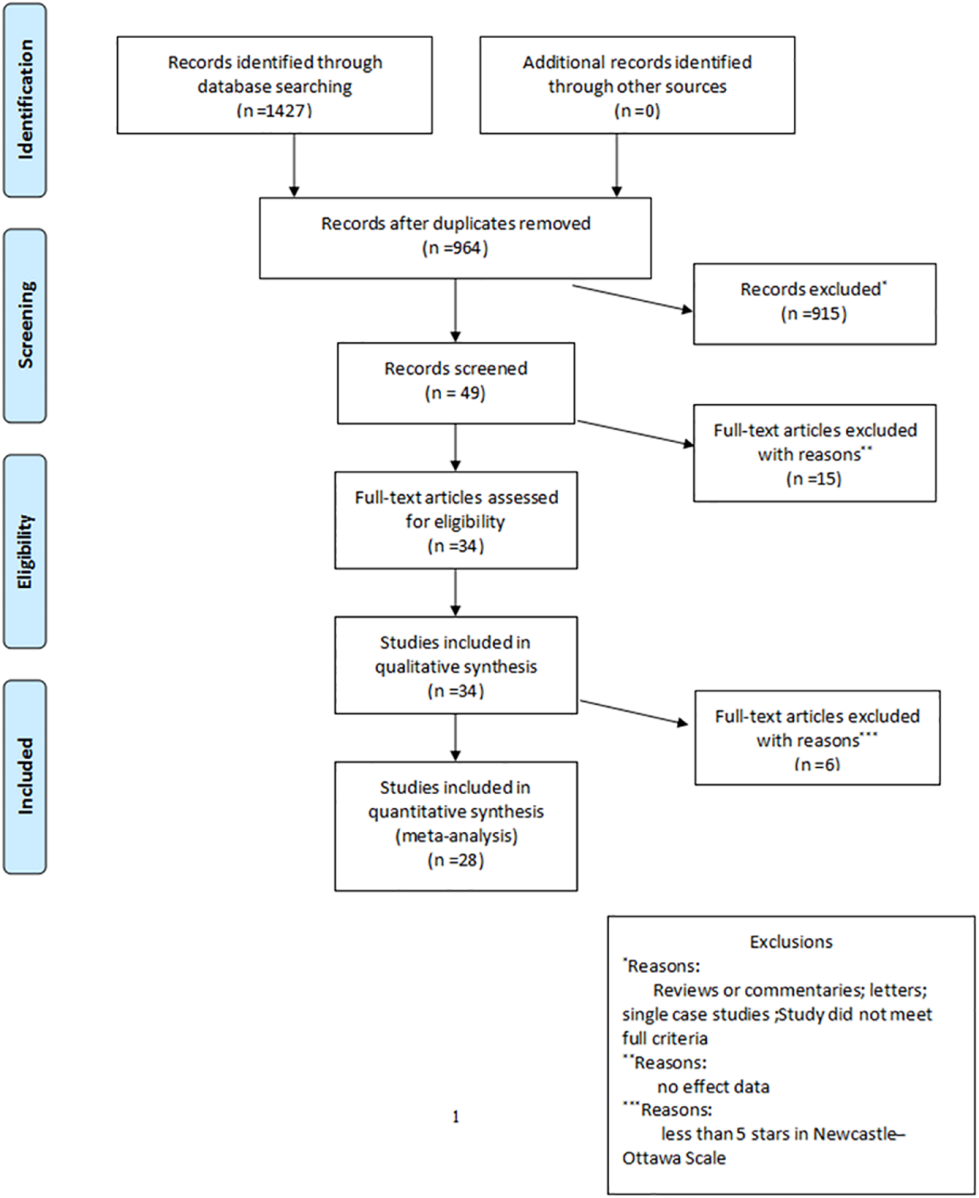
Flow chart of the literature search and article selection.

Fifteen studies were excluded because of a lack of available data, and 6 studies with fewer than 5 stars were excluded (S1 File). In total, 28 studies [2, 4, 6–31] that included 1774 subjects were retrieved for further assessment (Fig 1).

## Statistical methods

Risk ratios were calculated for the categorical data, and differences in the means were calculated for the continuous data. When the standard deviation (SD) and mean values were unavailable, the SD values were calculated using confidence intervals and P-values or were imputed from ranges and inter-quartile ranges [32], and the mean values were calculated from median values using the formulas outlined by Hozo et al. [33]. All these values are reported with 95% CIs. Statistical heterogeneity among the studies was assessed using Cochran's Q test and the I2 statistic. Studies with values of P<0.10 for the Q test or P>50% for the I2 statistic were considered statistically significant for heterogeneity and were assessed using random-effect models, whereas studies with non-significant values were assessed using fixed-effect models. To determine whether publication bias impacted the statistical results, a funnel plot was produced, and Egger's test was performed. For Egger's test, P<0.10 was considered statistically significant. All the statistical tests were two-sided. Stata version 10.0 and Review Manager were used to perform the data analysis.

## Results

The characteristics of the patients included in the trials and the NOS scores of the studies are summarized in Table 1. The major results of the current study are listed below.

**Table 1:** Characteristics of the studies included in the meta-analysis.

Author, year	Number of patients		Age		Gender		Tumor size		Follow-up (months)			P for the follow-up period	Recurrence		NOS score
	Lap	Open	Lap	Open	Lap (M:F)	Open (M:F)	Lap	Open	Lap	Open	Lap		Open		
Matthews 2002 [6]	21	12	53.9	50.5	13:8	8:4	4.5	4.9	20	18	N/A	0	1	6
Mochizuki 2006 [7]	12	10	60	59	6:6	4:6	2.7^t^	3.12^t^	26			N/A	0	0	5
Nishimura 2007 [4]	39	28	62	63	17:22	16:12	3.8^t^	4.2^t^	18.9	31.3	NS	1	4	6
Catena 2008 [10]	21	25	50.1	54.6	10:11	11:14	4.5	6.2	35	91	NS	0	1	6
Ishikawa 2006 [8]	14	7	61	67	6:8	4:3	2.9	8.5	60.2	61.3	N/A	2	1	6
Wu 2010 [11]	15	13	61.1	60.7	7:8	5:8	2.6	2.5	N/A	N/A	N/A	N/A	N/A	6
Pitsinis 2007 [9]	6	7	70^t^	68^t^	5:1	5:2	11.5^t^	5^t^	9			NS	0	0	6
Dai 2011 [13]	18	30	55	57	11:7	17:13	3.1	4.56	78^t^	64^t^	N/A	2	3	6
Karakousis 2011 [12]	40	40	67	70	26:14	23:17	3.6	4.3	28	43	N/A	1	1	6
Melstrom 2012 [13]	17	29	62	60	5:12	14:15	4.27	6.39	32	59	NS	0	4	6
Pucci 2012 [15]	57	47	62	66	30:27	22:25	3.8	9.2	N/A	N/A	N/A	N/A	N/A	6
Kim 2012 [16]	24	14	57.4	65.9	12:12	4:10	6.1	7.2	62.6^t^	58.3^t^	NS	1	3	5
Goh 2010 [2]	14	39	62^t^	64^t^	3:11	20:19	3.1^t^	4.5^t^	8^t^	21^t^	NS	0	2	5
Wan 2012 [17]	68	88	60.5^t^	58^t^	37:31	38:50	3.5^t^	4.0^t^	29^t^	36^t^	NS	3	4	6
De Vogelaere 2013 [18]	37	16	63.7	63.7	19:18	11:5	5.6	7.5	83^t^	71^t^	NS	0	6	6
Lee 2013 [19]	30	32	62	62	8:22	12:20	5.84	7.0	N/A	N/A	N/A	N/A	N/A	6
Shu 2013 [20]	15	21	54.21	52.37	8:7	11:10	N/A	N/A	N/A	N/A	N/A	N/A	N/A	6
Kasetsermwirjya 2014 [21]	23	10	69^t^	64^t^	8:15	6:4	2.9^t^	4.7^t^	46^t^	19^t^	NS	0	1	7
Kim 2014 [22]	156	250	59.75	58.73	55:101	102:148	3.45	5.46	42.9^t^			N/A	0	11	7
Cai 2015 [24]	90	66	58.6	56.8	31:59	29:37	3.5	4.3	21.0^t^	44.5^t^	N/A	6	8	7
de Angelis 2015 [25]	25	25	64.8	66.7	15:10	13:12	5.3	6.2	46.8			NS	1	2	5
Sista 2015 [29]	30	33	57.8	62.2	18:22	19:24	3.5^t^	6.1^t^	35^t^	67^t^	NS	3	8	7
Yan 2015 [31]	158	68	57	56.5	68:90	29:39	4.5	5.0	32^t^			NS	N/A	N/A	6
Lin 2014 [23]	23	23	63.4	62	12:11	7:16	7.2	7.3	34^t^			N/A	1	2	6
Hsiao 2015 [26]	18	21	66.6	64.5	8:10	7:14	6.3	6.0	3.1(year)	5.6(year)	NS	1	0	7
Piessen 2015 [27]	224	224	N/A	N/A	111:113	109:115	N/A	N/A	N/A	N/A	N/A	N/A	N/A	7
Takahashi 2015 [28]	12	15	64	66	7:5	10:5	5.5	7.7	57^t^	69^t^	N/A	1	2	7
Xue 2015 [30]	55	112	60.9	59.9	26:29	46:66	4,1	5.6	25^t^	47^t^	N/A	0	3	5

Value is expressed as the mean unless otherwise indicated.

^t^: median value. Lap: laparoscopic; Open: open resection; NOS: Newcastle-Ottawa scoring system; N/A: not available; NS: not significant.

### 1. Operative outcomes

**Fig 2:**
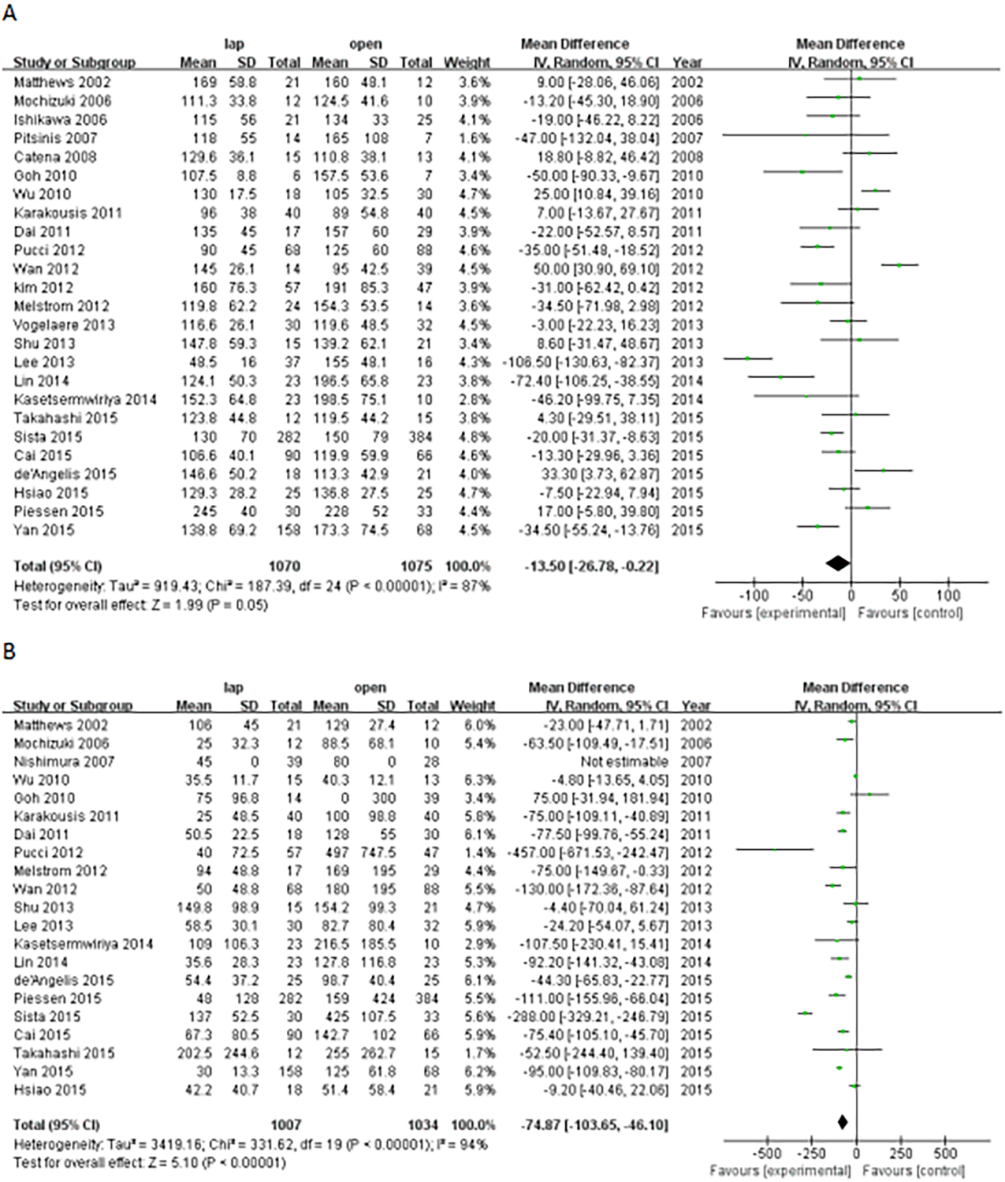
Forest plot for the operative outcomes (A: operation time; B: blood loss).

The surgical time of the LAP group patients was shorter than that of the OPEN group patients (WMD, -13.50 min; 95% CI, -26.78 to -0.22; P = 0.05). In total, 21 studies presented comparisons between LAP group patients and OPEN group patients, and the results showed that the patients in the LAP group had reduced blood loss (WMD, -74.87 ml; 95% CI, -103.65 to -46.1; P<0.0001; Fig 2).

### 2. Short-term postoperative outcomes

**Fig 3:**
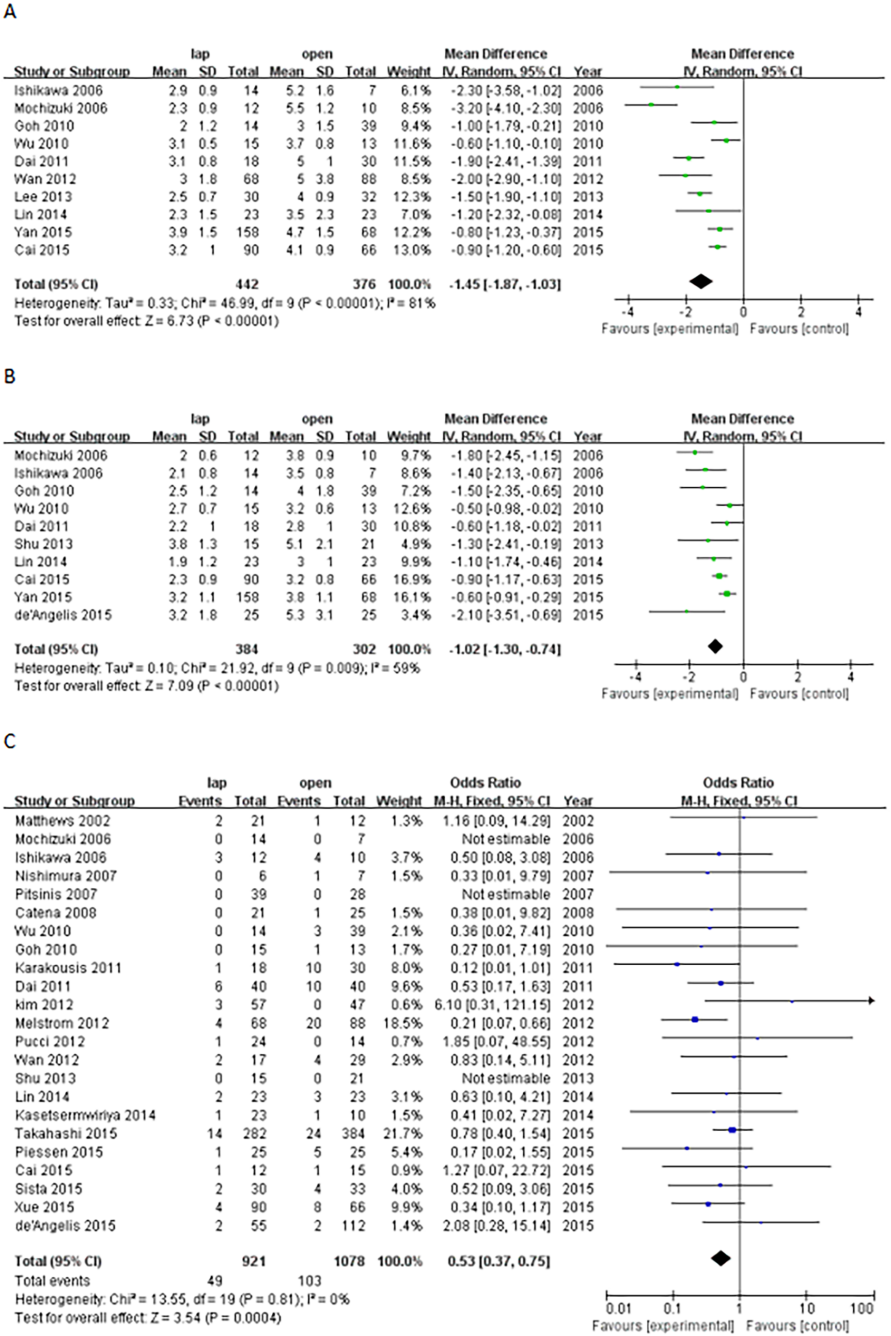
Forest plot for the short-term postoperative outcomes (A: postoperative time to oral intake; B: postoperative time to first flatus; C: postoperative complications).

The postoperative time to oral intake (WMD, -1.45 days; 95% CI, -1.87 to -1.03; P<0.0001) and postoperative time to first flatus (WMD, -1.02 days; 95% CI, -1.30 to -0.74; P<0.0001) favored the patients in the LAP group. The number of postoperative hospital days was 3.16 days shorter for the LAP group than the OPEN group (WMD, -3.16 days; 95% CI, -3.85 to 2.48; P<0.0001). The overall rate of postoperative complications was reduced in the LAP group (odds ratio (OR), 0.53; 95% CI, 0.37 to 0.75; P<0.01; Fig 3).

### 3. Oncological outcomes

**Fig 4:**
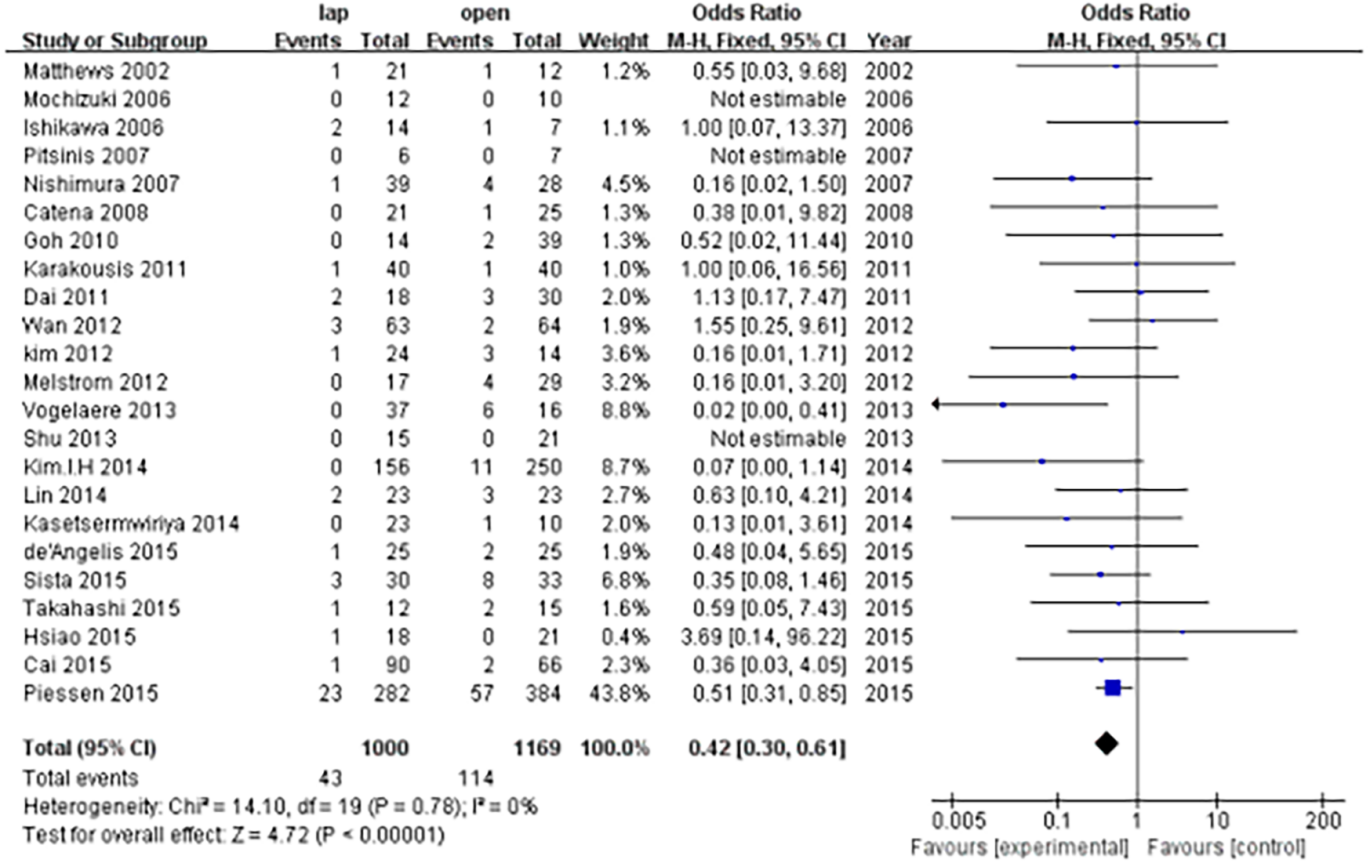
Forest plot for the oncological outcomes (recurrence).

The recurrence risk was 4.3% in the LAP group and 9.75% in the OPEN group. Thus, patients who underwent LAP were less likely to experience recurrence compared with the patients who underwent OPEN (OR, 0.42; 95% CI, 0.30 to 0.61; P<0.001; Fig 4).

### 4. Survival

**Fig 5:**
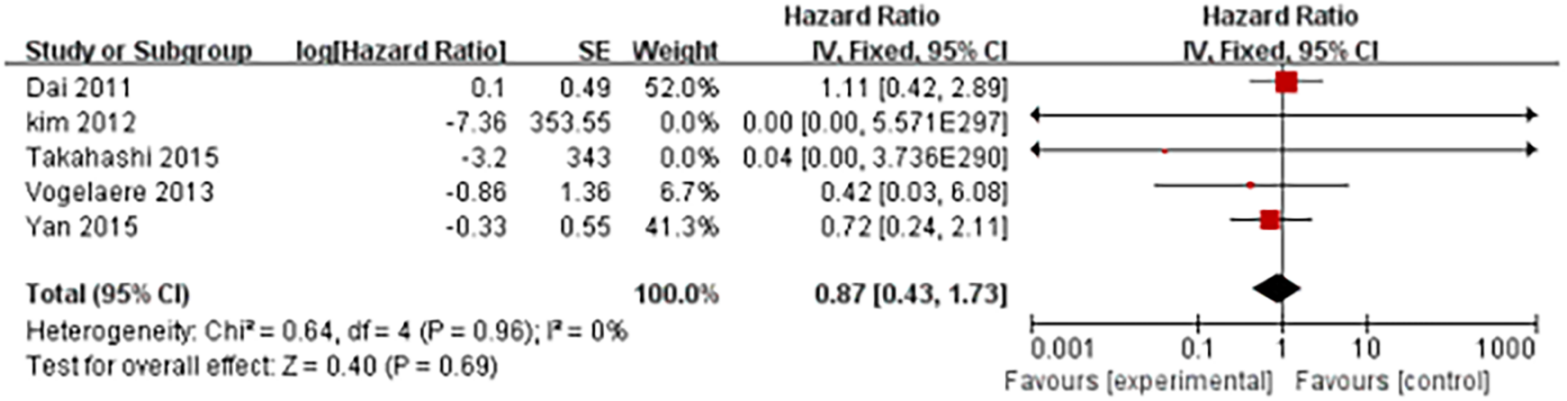
Forest plot for survival (overall survival).

The long-term follow-up results did not indicate significant differences between the two groups of patients regarding the recurrence-free survival (RFS) rate (hazard ratio (HR), 0.88; 95% CI, 0.39 to 2.00; P = 0.77) and the overall survival (OS) rate (HR, 0.87; 95% CI, 0.43–1.73; P = 0.69; Fig 5).

### 5. Subgroup analysis of tumor size (<5 cm vs. >5 cm)

**Fig 6:**
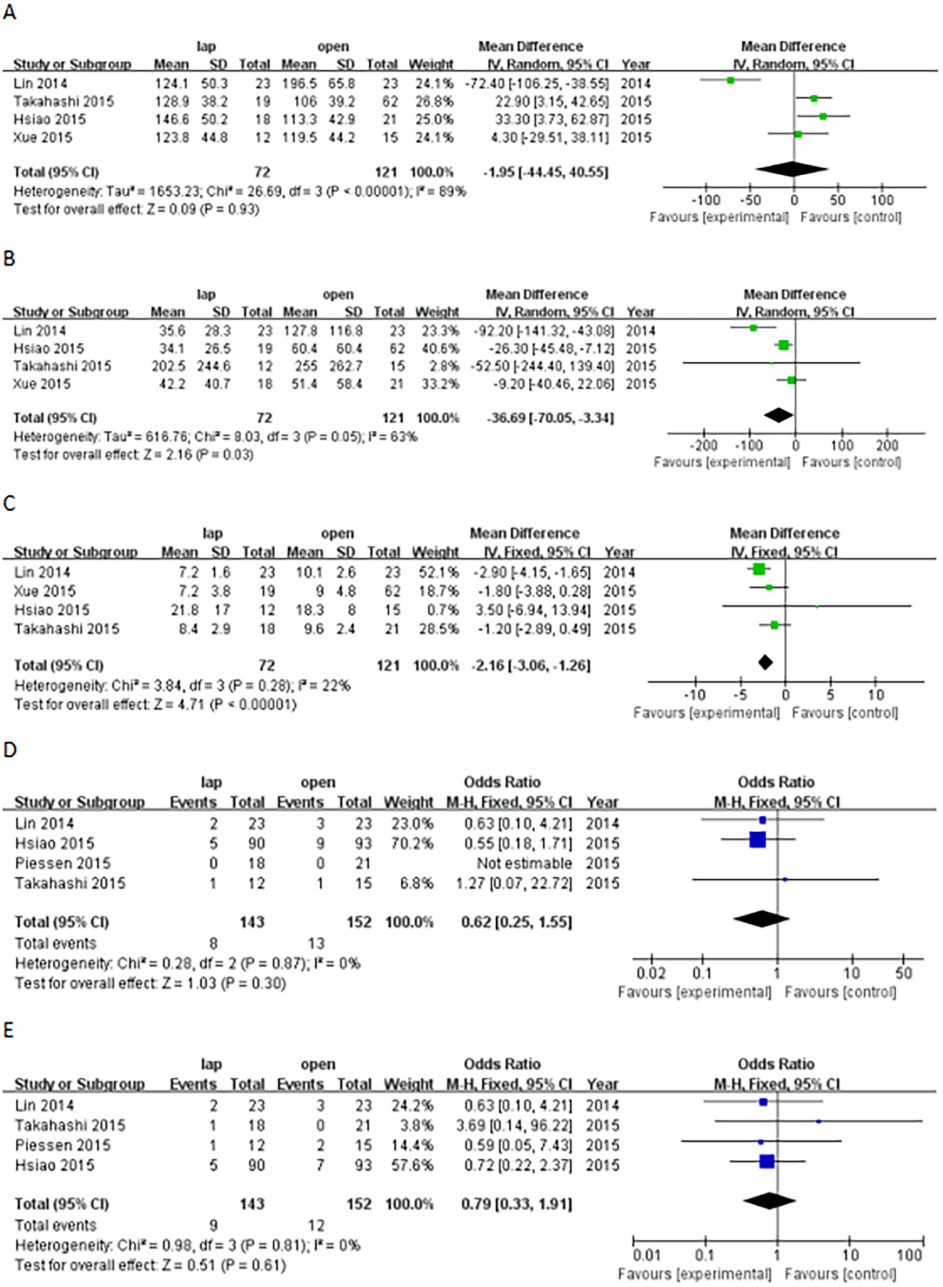
Forest plot for the subgroup of tumors larger than 5 cm (A: operation time; B: blood loss; C: hospital stay; D: postoperative complications; E: recurrence).

For tumors larger than 5 cm, the comparative analysis did not indicate significant differences in operation time (WMD, -1.95 min; 95% CI; -44.45 to 40.55; P = 0.93), postoperative complications (OR, 0.62; 95% CI, 0.25–1.55; P = 0.30), or recurrence rate (OR, 0.79; 95% CI, 0.33–1.91; P = 0.61) between the LAP group and OPEN group patients, though LAP procedure was associated with reduced blood loss (WMD, -36.69 ml; 95% CI, -70.05 to -3.34; P = 0.03) and shorter hospital stays (WMD, -2.16 days; 95% CI, -3.06 to -1.26; P<0.01; Fig 6).

### 6. Publication bias

**Fig 7:**
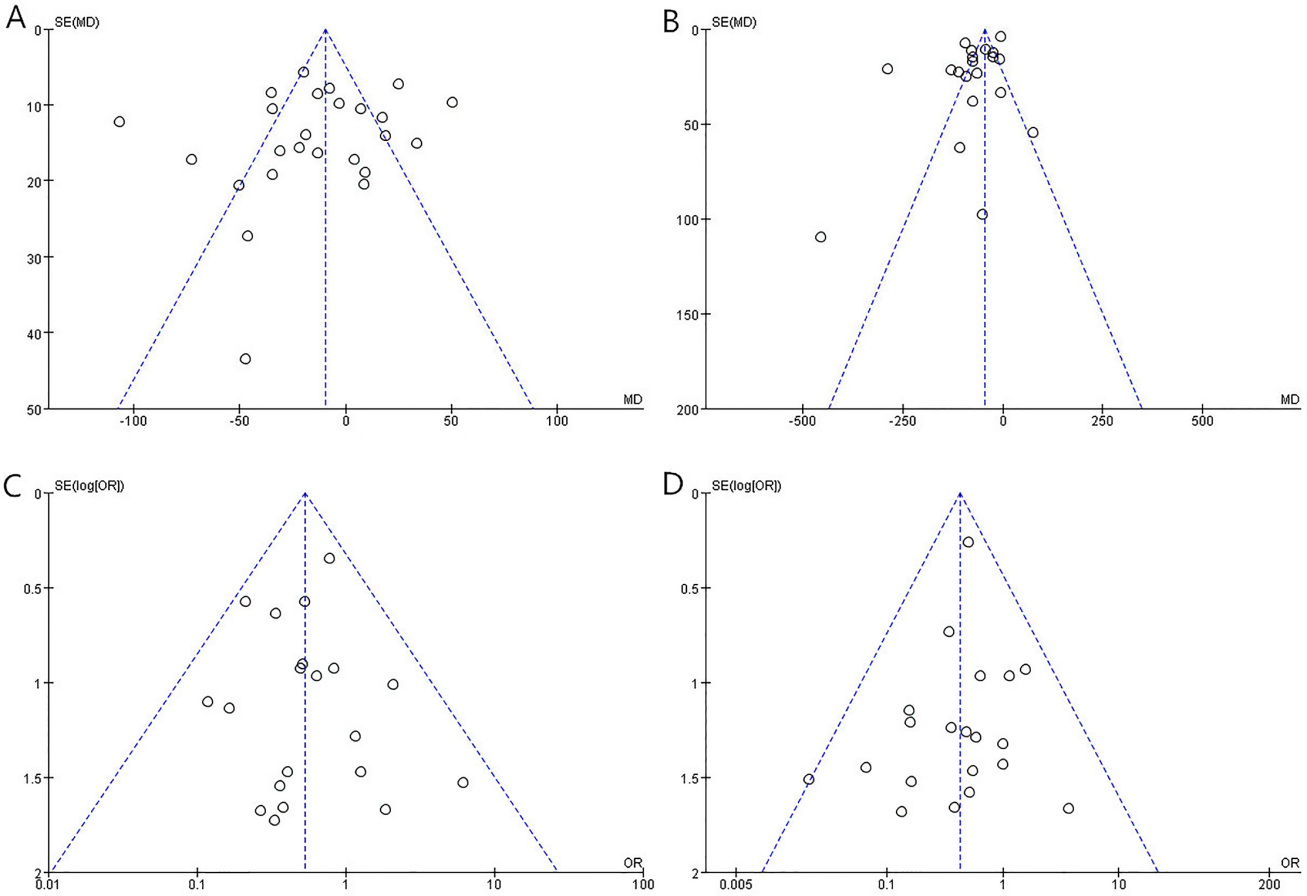
Funnel plot for publication bias (A: operation time; B: blood loss; C: postoperative complications; D: recurrence).

A funnel plot analysis and Begg’s regression asymmetry test were performed when more than 10 studies were compared; however, no evidence of publication bias was observed (Fig 7).

## Discussion

Compared with open surgery, laparoscopic gastric resection presents the potential advantages of smaller incisions and reduced bowel manipulation. When choosing an operation method, the surgeon’s first concern is safety. Our data demonstrated that the LAP group experienced reduced blood loss (P<0.0001) and surgical time compared with the OPEN group. This finding is consistent with the results of most published studies [24, 32]. There are potential advantages to decreased blood loss, such as reducing the need for blood transfusion during the operation, stabilizing intraoperative vital signs and benefiting the patient’s postoperative recovery. The LAP procedure offers a clear view of the vessel, nerve and fascia structures, thereby facilitating more careful surgical procedures and reducing the likelihood of damage to nearby structures. Shorter surgical times are believed to reduce the likelihood of infection and can increase patients’ peace of mind regarding the surgery. According to the meaning of enhanced recovery program after surgery, LAP is no doubt a better choice. Differences can be attributed to making the laparotomy, closing longer incisions or suturing the gastrotomy defect, which are more likely with the open versus laparoscopic technique (where staple devices may be more commonly used). When faced with a difficult situation that may cost a lot of time, OPEN surgery is preferred. As surgeons become more proficient, the surgical time will decrease. The learning curve for laparoscopic gastric GIST surgery requires approximately 40 cases [17]. The time of the LAP procedure will be reduced in the future, as the technology is learned and improved upon by more young surgeons. However, the mean time for the LAP group was 129.4 min, whereas that of the OPEN group was 144.7 min, and no statistical significance was detected when comparing the LAP group’s 13.5-min shorter operative time with that of the OPEN group. Unlike our results, in the meta-analysis by Pelletier et al. [34], no difference in operative time and blood loss over time with LAP was found. However, their study included only 267 patients, which was far fewer than the present study.

We found that the LAP group exhibited enhanced short-term postoperative outcomes, including postoperative time to oral intake (P<0.0001) and postoperative time to first flatus (P<0.0001) [4, 32, 35]. Moreover, the LAP group exhibited shorter hospital stays (P<0.0001) and fewer postoperative complications (P<0.01). The significant benefit of LAP is its smaller incision, which results in less pain and bed rest, thus accelerating recovery. The clearer operative field of the LAP procedure has the potential to reduce complications, including peritonitis or poor wound healing, because the operation quality can be controlled more easily.

Our data demonstrated that patients in the LAP group were less likely than OPEN patients to experience recurrence (4.3% vs. 9.75%, P<0.001). This difference was likely because open resection is more likely to be selected for large tumors, which are more likely to recur. Therefore, LAP is generally used to treat small tumors, which typically exhibit lower recurrence. The size of the tumor is thought to be an important factor when evaluating the risk of recurrence. Various classification systems for predicting recurrence of GIST have been proposed. However, the tumor size is taken into consideration when calculating the risk of recurrence using the system by the National Institute of Health, the Modified National Institute of Health (NIH 2008), or the Armed Forces Institute of Pathology (AFIP), the prognostic nomogram by Memorial Sloan Kettering Cancer Center (MSKCC) or contour maps, In other words, it might be the bulky tumor itself, rather than the operation method, that leads to recurrence. Pelletier et al. found less recurrence in patients who underwent LAP [34], and these authors thought the difference was due to the shorter follow-up time this group. Therefore, the bias of follow up time also could be a factor that affects the recurrence rate. However, we found no significant difference in follow-up time between the LAP and OPEN groups. In terms of the recurrence site, there were 5 recurrences after a median of 26.1 months of follow-up (one in the LAP group and 4 in the OPEN group), including two instances of local recurrence (one in the LAP group and one in the OPEN group), one peritoneal recurrence (OPEN group), and two liver metastasis cases (OPEN group) in the study by Nishimura et al. [4]. In Ishikawa et al.’s study [8], one local recurrence and one liver recurrence occurred in LAP group, while one local recurrence occurred in OPEN group. Because most research did not address recurrence, we could not determine whether the surgical technique had an impact on oncologic outcomes. However, the long-term follow-up results confirmed that there were no significant differences in the long-term survival of the two groups, indicating that LAP can remove the tumor as cleanly as OPEN and does not change the long-term results. The NCCN guidelines also recommend the use of LAP for tumors smaller than 5 cm, although the guidelines do not provide information for tumors larger than 5 cm [35].

To determine the most effective surgical procedures for tumors larger than 5 cm, we performed a subgroup analysis based on tumor size. In the extracted data, the operation time of the LAP group patients was not significantly shorter than that of the OPEN group when the tumor size was >5 cm. Fully exposing large tumors is difficult; therefore, removing these tumors presents an increased chance of involving adjacent tissue. Moreover, large tumors are fragile and have a rich blood supply, making them more difficult to remove via laparoscopy and requiring additional surgical time. Lin et al.indicated that the LAP group had shorter surgery times than the OPEN group in a study of 46 pair-matched patients with tumors larger than 5 cm in diameter [23]. However, Goh et al. found that a major limitation of retrospective studies is selection bias [2]. These authors matched patients based on tumor location and resection and found that the LAP group was associated with significantly longer operating times than the OPEN group. Therefore, proper controls are required to perform these comparisons.

No significant differences in postoperative complications, recurrence rates and long-term disease-free survival rate were observed between the two groups when the tumor was larger than 5 cm. However, for large tumors, the LAP procedure was associated with favorable results in terms of blood loss (P = 0.03) and hospital stay (P<0.01), indicating that LAP is associated with favorable short-term outcomes without compromising oncological outcomes. Hsiao et al. reported the same conclusions [26]. ESMO (2012) discourages the use of LAP for GISTs larger than 5 cm because of the increased risk of rupture, which results in dissemination; however, an increasing number of studies in recent years have reported that large GISTs have been successfully removed without rupture [13]. Nonetheless, avoiding rupture should be a primary concern. A surgeon’s experience and skill must be considered prior to selecting the LAP procedure. Severino et al.’s study focused on the efficiency of LAP for large and small GISTs [36]. They found that LAP was safe for large GISTs, which is consistent with our study. We believe that GISTs larger than 5 cm should not be a contraindication for tumor removal with LAP. However, tumors that are too large for laparoscopy should be removed via open resection to increase the efficacy of the treatment. Hsiao et al. proposed that 8 cm should be the upper limit for LAP, whereas other studies have indicated that open resection remains the best surgical option for GISTs larger than 10 cm [17, 26]. Further studies should be performed to validate these findings.

Tumor position should be considered when considering the optimal operative method. However, no subgroup analysis of tumor site was conducted in this study because of insufficient data. The NCCN recommends that GISTs located in the greater curvature [35], the anterior wall and the jejuno-ileum can be removed via LAP by an experienced surgeon. Xue et al. showed that performing LAP in the greater curvature and anterior wall resulted in reduced blood loss and hospital stays [30]. This difference was likely related to the occurrence of shallow tumors in these two locations and the wider operating space, which facilitated the localization and excision of these tumors. The gastroesophageal junction is a controversial position in terms of LAP use. Nguyen et al. performed LAP procedures on 43 patients [37], and three conversions were caused by adherence or a position near the GE junction. For tumors located near the pylorus, the cardia or the lesser curvature, obstructions are the most common problem for the LAP procedure. However, comparisons of the safety and effectiveness of the LAP and OPEN procedures at these sites are rare, which increases the difficulty of determining the best method. Before performing additional random control trials, surgeons should choose an operative solution based on their experience. Endoscopy-assisted laparoscopic surgery plays an important role in certain cases, especially for intragastric and small tumors.

A limitation of this study is the insufficient number of studies on large GISTs. Therefore, the results of the subgroup analysis should be evaluated by high-quality randomized controlled trials with larger sample sizes that compare the OPEN and LAP procedures for GISTs. It cannot be denied that there was also selection bias between the LAP and OPEN groups.

It is believed that the tumor location and size should not be the only factors that influence the decision regarding the surgical procedure. Indeed, additional tumor parameters and operator proficiency should also be considered. Therefore, detailed preoperative examinations are necessary. Regardless of the procedure selected, a complete excision, smooth operating conditions and rupture avoidance are important factors for successful surgery.

## Supporting information

S1 File Excluded full-text articles and the reasons. (DOCX)

S1 Fig PRISMA 2009 checklist. (DOC)
