# Supplementary material for: Meta-analysis of laparoscopic vs. open resection of gastric gastrointestinal stromal tumors
Source: PLoS One. 2017 May 9;12(5):e0177193. doi: 10.1371/journal.pone.0177193 (PMC5423634; doi:10.1371/journal.pone.0177193)
Supplement: S1 File — (DOCX) [file pone.0177193.s001.docx]

In our study, 1427 publications were identified using the predefined search strategy.(Embace 682,Pubmed 325,Web of science 463). 463 were excluded because of duplicates .After we reviewed the title and abstract , 930 studies were further excluded ,included 757 irrelevant studies,25 reviews,133 studies were non-comparative,15 studies were without available data.

1. Passage excluded because of low quality(6):[1-6]
2. Passage included(28): [7-34]

**References:**

1. Chen Y, Liu K, Yeh C, Hsu J, Liu Y, Tsai C, et al. Laparoscopic Resection of Gastrointestinal Stromal Tumors: Safe, Efficient, and Comparable Oncologic Outcomes. J Laparoendosc Adv S. 2012; 8: 758-763.

2. Bellorin O, Kundel A, Ni M, Litong D. Surgical Management of Gastrointestinal Stromal Tumors of the Stomach. JSLS : Journal of the Society of Laparoendoscopic Surgeons. 2014; 1: 46-49.

3. Schwameis K, Fochtmann A, Schwameis M, Asari R, Schur S, Köstler W, et al. Surgical treatment of GIST – An institutional experience of a high-volume center. Int J Surg. 2013; 9: 801-806.

4. Poskus E, Petrik P, Petrik E, Lipnickas V, Stanaitis J, Strupas K. Surgical management of gastrointestinal stromal tumors: a single center experience. Wideochir Inne Tech Maloinwazyjne. 2014; 1: 71-82.

5. Silberhumer GR, Hufschmid M, Wrba F, Gyoeri G, Schoppmann S, Tribl B, et al. Surgery for Gastrointestinal Stromal Tumors of the Stomach. J Gastrointest Surg. 2009; 7: 1213-1219.

6. Orsenigo E, Gazzetta P, Palo SD, Tamburini A, Staudacher C. Experience on surgical treatment of gastrointestinal stromal tumor of the stomach. Updates in Surgery. 2010; 2: 101-104.

7. Matthews BD, Walsh RM, Kercher KW, Sing RF, Pratt BL, Answini GA, et al. Laparoscopic vs open resection of gastric stromal tumors. Surgical Endoscopy. 2002; 5: 803-807.

8. Mochizuki Y, Kodera Y, Fujiwara M, Ito S, Yamamura Y, Sawaki A, et al. Laparoscopic Wedge Resection for Gastrointestinal Stromal Tumors of the Stomach: Initial Experience. Surg Today. 2006; 4: 341-347.

9. Ishikawa K, Inomata M, Etoh T, Shiromizu A, Shiraishi N, Arita T, et al. Long-term outcome of laparoscopic wedge resection for gastric submucosal tumor compared with open wedge resection. Surg Laparosc Endosc Percutan Tech. 2006; 2: 82-85.

10. Nishimura J, Nakajima K, Omori T, Takahashi T, Nishitani A, Ito T, et al. Surgical strategy for gastric gastrointestinal stromal tumors: laparoscopic vs. open resection. Surgical Endoscopy. 2007; 6: 875-878.

11. Pitsinis V, Khan AZ, Cranshaw I, Allum WH. Single center experience of laparoscopic vs. open resection for gastrointestinal stromal tumors of the stomach. Hepatogastroenterology. 2007; 74: 606-608.

12. Catena F, Di Battista M, Fusaroli P, Ansaloni L, Di Scioscio V, Santini D, et al. Laparoscopic Treatment of Gastric Gist: Report of 21 Cases and Literature’s Review. J Gastrointest Surg. 2008; 3: 561-568.

13. Wu J, Yang C, Wang M, Wu M, Lin M. Gasless Laparoscopy-Assisted Versus Open Resection for Gastrointestinal Stromal Tumors of the Upper Stomach: Preliminary Results. J Laparoendosc Adv S. 2010; 9: 725-729.

14. Goh BKP, Chow PKH, Chok A, Chan W, Chung YA, Ong H, et al. Impact of the Introduction of Laparoscopic Wedge Resection as a Surgical Option for Suspected Small/Medium-Sized Gastrointestinal Stromal Tumors of the Stomach on Perioperative and Oncologic Outcomes. World J Surg. 2010; 8: 1847-1852.

15. Karakousis GC, Singer S, Zheng J, Gonen M, Coit D, DeMatteo RP, et al. Laparoscopic Versus Open Gastric Resections for Primary Gastrointestinal Stromal Tumors (GISTs): A Size-Matched Comparison. Ann Surg Oncol. 2011; 6: 1599-1605.

16. Dai QQ, Ye ZY, Zhang W, Lv ZY, Shao QS, Sun YS, et al. [Laparoscopic versus open wedge resection for gastrointestinal stromal tumors of the stomach: a clinical controlled study]. Zhonghua Wei Chang Wai Ke Za Zhi. 2011; 8: 603-605.

17. Melstrom LG, Phillips JD, Bentrem DJ, Wayne JD. Laparoscopic Versus Open Resection of Gastric Gastrointestinal Stromal Tumors. American Journal of Clinical Oncology. 2012; 5: 451-454.

18. Pucci MJ, Berger AC, Lim P, Chojnacki KA, Rosato EL, Palazzo F. Laparoscopic approaches to gastric gastrointestinal stromal tumors: an institutional review of 57 cases. Surgical Endoscopy. 2012; 12: 3509-3514.

19. Kim K, Kim M, Jung G, Kim S, Jang J, Kwon H. Long term survival results for gastric GIST: is laparoscopic surgery for large gastric GIST feasible? World J Surg Oncol. 2012; 1: 230.

20. Wan P, Yan C, Li C, Yan M, Zhu ZG. Choices of Surgical Approaches for Gastrointestinal Stromal Tumors of the Stomach: Laparoscopic versus Open Resection. Digest Surg. 2012; 3: 243-250.

21. De Vogelaere K, Hoorens A, Haentjens P, Delvaux G. Laparoscopic versus open resection of gastrointestinal stromal tumors of the stomach. Surgical Endoscopy. 2013; 5: 1546-1554.

22. Lee P, Lai P, Yang C, Chen C, Lai I, Lin M. A gasless laparoscopic technique of wide excision for gastric gastrointestinal stromal tumor versus open method. World J Surg Oncol. 2013; 1: 44.

23. Shu Z, Sun L, Li J, Li Y, Ding D. Laparoscopic versus open resection of gastric gastrointestinal stromal tumors. Chinese J Cancer Res. 2013; 2: 175-182.

24. Kasetsermwiriya W, Nagai E, Nakata K, Nagayoshi Y, Shimizu S, Tanaka M. Laparoscopic Surgery for Gastric Gastrointestinal Stromal Tumor Is Feasible Irrespective of Tumor Size. J Laparoendosc Adv S. 2014; 3: 123-129.

25. Kim I, Kim I, Kwak S, Kim SW, Chae H. Gastrointestinal stromal tumors (GISTs) of the stomach: a multicenter, retrospective study of curatively resected gastric GISTs. Annals of Surgical Treatment and Research. 2014; 6: 298.

26. Lin J, Huang C, Zheng C, Li P, Xie J, Wang J, et al. Laparoscopic versus open gastric resection for larger than 5 cm primary gastric gastrointestinal stromal tumors (GIST): a size-matched comparison. Surgical Endoscopy. 2014; 9: 2577-2583.

27. Cai J, Chen K, Mou Y, Pan Y, Xu X, Zhou Y, et al. Laparoscopic versus open wedge resection for gastrointestinal stromal tumors of the stomach: a single-center 8-year retrospective cohort study of 156 patients with long-term follow-up. Bmc Surg. 2015; 1: 58.

28. de Angelis N, Brunetti F, Felli E, Mehdaoui D, Memeo R, Carra MC, et al. Laparoscopic Versus Open Gastric Wedge Resection for Primary Gastrointestinal Tumors. Surgical Laparoscopy, Endoscopy & Percutaneous Techniques. 2015; 2: 143-146.

29. Hsiao C, Yang C, Lai I, Chen C, Lin M. Laparoscopic resection for large gastric gastrointestinal stromal tumor (GIST): intermediate follow-up results. Surgical Endoscopy. 2015; 4: 868-873.

30. Piessen G, Lefèvre JH, Cabau M, Duhamel A, Behal H, Perniceni T, et al. Laparoscopic Versus Open Surgery for Gastric Gastrointestinal Stromal Tumors. Ann Surg. 2015; 5: 831-840.

31. Takahashi T, Nakajima K, Miyazaki Y, Miyazaki Y, Kurokawa Y, Yamasaki M, et al. Surgical Strategy for the Gastric Gastrointestinal Stromal Tumors (GISTs) Larger Than 5 cm. Surgical Laparoscopy, Endoscopy & Percutaneous Techniques. 2015; 2: 114-118.

32. Sista F, Pessia B, Abruzzese V, Cecilia EM, Schietroma M, Carlei F, et al. Twelve years of gastric GIST A retrospective study of laparoscopic and open approach. Ann Ital Chir. 2015; 4: 349-356.

33. Xue A, Fu Y, Gao X, Fang Y, Shu P, Lin J, et al. [Comparative study of laparoscopic and open surgery for gastric gastrointestinal stromal tumors]. Zhonghua Wei Chang Wai Ke Za Zhi. 2015; 11: 1119-1123.

34. Yan P, Liu J, Hu X, Liu J, Wu Y, Zhao Y, et al. [Clinical efficacy comparison between laparoscopic and open surgery in the treatment of gastric gastrointestinal stromal tumor]. Zhonghua Wei Chang Wai Ke Za Zhi. 2015; 8: 808-811.
